# Candida albicans: factor agravante en pacientes con covid-19

**DOI:** 10.21142/2523-2754-1004-2022-132

**Published:** 2023-12-26

**Authors:** Daniela San Martín Andrade, Cristopher Andrés Cárdenas Amendaño, Allison Brigitte Solórzano Cuenca, Johanna Maribel Ulloa Pacheco, Priscilla Medina-Sotomayor

**Affiliations:** 1 Carrera de Odontologia de la Universidad Catolica de Cuenca. Campus Universitario Azogues, Ecuador. daniela.sanmartin@ucacue.edu.ec, ipmedinas@ucacue.edu.ec Universidad Católica de Cuenca Carrera de Odontologia Universidad Catolica de Cuenca Campus Universitario Azogues Ecuador daniela.sanmartin@ucacue.edu.ec ipmedinas@ucacue.edu.ec; 2 Universidad Catolica de Cuenca. Campus Universitario Azogues, Ecuador. cristopher.cardenas@est.ucacue.edu.ec, asolorzanoc02@est.ucacue.edu.ec, jmulloap18@est.ucacue.edu.ec Universidad Católica de Cuenca Universidad Catolica de Cuenca Campus Universitario Azogues Ecuador cristopher.cardenas@est.ucacue.edu.ec asolorzanoc02@est.ucacue.edu.ec jmulloap18@est.ucacue.edu.ec

**Keywords:** candidiasis oral, coronavirus, COVID-19, oral candidiasis, coronavirus, COVID-19

## Abstract

El hongo *Candida albicans* puede provocar en nuestro organismo diversos cuadros de afección que dependen directamente de la condición sistémica del huésped. Pacientes con COVID-19 que presentan previamente este hongo tienen la capacidad de aumentar la probabilidad de morbilidad y mortalidad, ya que este puede ubicarse en zonas correspondientes al sistema respiratorio y provocar un déficit de sus funciones. Si no es tratado a tiempo, puede proliferar y dirigirse al sistema sanguíneo y el digestivo. Muchos de los pacientes con problemas respiratorios asociados con esta causa necesitan de ventilación mecánica para combatir la afección.

**Objetivo:**

Relacionar la presencia de *Candida albicans* como un factor agravante en pacientes con COVID-19.

**Materiales y métodos:**

Se realizó una revisión bibliográfica en las bases de datos Redalyc, SciELO, PubMed, ResearchGate, Science Direct y Google Académico. Los criterios de inclusión utilizados fueron: artículos en inglés y español, además desde 2020 hasta la fecha. Se analizaron 65 artículos científicos que cumplieron los criterios de búsqueda y se pudo determinar que la candidiasis oral afecta de manera negativa a pacientes con infección por COVID-19, lo que aumenta el riesgo de que necesiten ingresar a UCI para hacer uso de ventiladores artificiales.

## Abstract (en)

The Candida Albicans fungus in our body can cause various conditions and will depend directly on the systemic condition of the host. Patients with COVID-19 who have previously presented this fungus can increase the likelihood of morbidity and mortality, since this microorganism can be located in areas that correspond to the respiratory system, generating a functional deficit. If not treated timely, it will proliferate into the blood and digestive system. Many patients with respiratory difficulties on account of this condition require mechanical ventilation to combat it.

**Objective:**

To relate the presence of Candida Albicans as an aggravating factor in patients with COVID-19.

**Materials and:**

**methods:** A literature review took place using the Redalyc, Scielo, PubMed, Research gate, Science direct, Google Scholar databases. The inclusion criteria used were: articles in English and Spanish, along with articles published from 2020 up to date. There were 65 scientific articles that met the search criteria and were analyzed. The analysis determined that oral candidiasis negatively affects patients with COVID-19 infection, increasing the risk of admission to the ICU with the use of artificial ventilators.

## INTRODUCCIÓN

El coronavirus (COVID-19) es una enfermedad altamente contagiosa producida por el virus SARS CoV-2, responsable de la aparición de diversa sintomatología, siendo las alteraciones respiratorias las más recurrentes, principalmente en cuadros graves de la enfermedad ^1^. En ciertas ocasiones, los cuadros clínicos empeoran debido a la aparición de infecciones secundarias o patógenos oportunistas adquiridos. La incidencia de infecciones por hongos ha aumentado significativamente, lo que contribuye a la mortalidad y morbilidad ^1^.

*Candida albicans* es un hongo oportunista que pueden aparecer en el cuerpo humano teniendo cierta predisposición sobre la cavidad oral, sobre todo en pacientes portadores de prótesis removibles, lo que causa infecciones a nivel de la mucosa, que sin el tratamiento adecuado pueden ser una fuente de infección potencialmente mortal para el ser humano ^2^.

Los pacientes portadores de prótesis removibles pueden presentar placas rojizas o blanquecinas en la cavidad oral a causa de la presencia de *Candida albicans*. Su posible etiología radica en el estrés, la higiene bucal deficiente, la xerostomía, las prótesis en mal estado, la terapia antibiótica o la administración de corticoides. 

Se ha reportado que pacientes portadores de prótesis dental con un sistema inmunitario débil y que han adquirido el virus del SARS-COV-2 pueden dar paso a una infección aguda por la presencia de *Candida albicans*. Si al deterioro general de la salud y los problemas respiratorios agudos en estos pacientes se suma la presencia de este hongo, puede aumentar la tasa de morbilidad y mortalidad ^2^.

Según reportan ciertos estudios, la tasa de mortalidad de los infectados con COVID-19 era de un 50,47%, de los cuales se descubrió que el 80% de los fallecidos tenían presencia de hongos en su organismo. Además, los pacientes coinfectados permanecieron hospitalizados por más tiempo y tuvieron mayores probabilidades de morir. El riesgo de muerte se vio incrementado por coinfecciones bacterianas y micóticas, pero se logró detectar un mayor riesgo de muerte en los casos de *Candida albicans* y *Pseudomona* spp. ^2^.

Por esta razón, el objetivo de la presente revisión bibliográfica es relacionar la presencia de *Candida albicans* como un factor agravante en pacientes con COVID-19.

## MATERIALES Y MÉTODOS

Se realizó una revisión de literatura para desarrollar un análisis crítico reflexivo del contenido de artículos originales con la revisión de las bases de datos SciELO, PubMed, Google Académico y Science Direct desde enero de 2020. El objetivo planteado para el presente estudio es relacionar la presencia de *Candida albicans* como un factor agravante en pacientes con COVID-19.

La estrategia de búsqueda utilizó descriptores y palabras claves conectados a través de operadores booleanos AND y OR vinculados con el título del artículo, resumen y palabras clave. Los descriptores o términos claves utilizados fueron “COVID-19”, “coronavirus”, “lesiones orales”, “candidiasis”, “*Candida albicans*”, identificadas a través DeCS (Descriptores en Ciencias de la Salud) y MeSH (Medical Subject Headings).

Los criterios de inclusión para la selección de documentos fueron “artículos” y “revisiones” en español e inglés, con acceso a texto completo publicados entre 2020 y 2021. Se excluyeron aquellos que no relacionan la *Candida albicans* como factor agravante de la COVID-19. 

Finalmente se realizó una búsqueda manual con el fin de rescatar otros estudios potencialmente relevantes. 

## RESULTADOS Y DISCUSIÓN

La COVID-19 es una enfermedad infecciosa en donde la mayor parte de infectados presentan problemas respiratorios. Ciertos pacientes tienen la capacidad de mostrar una evolución favorable frente a esta afección sin ningún tratamiento en específico; no obstante, pacientes con alto riesgo sistémico, como hipertensión, diabetes o cáncer, el cuadro clínico puede variar por la cual es importante una atención inmediata ^3^.

Este virus puede transmitirse de una persona infectada a través de la boca o la nariz por pequeñas partículas de líquido expulsadas mediante expectoración durante el habla o los estornudos. Estas partículas van desde pequeñas gotas visibles hasta aerosoles de tamaños muy reducido ^4^.

Los síntomas más tempranos varían, aunque por lo general incluyen fiebre, tos, dolor de garganta, pérdida del gusto y el olfato, disnea y, en situaciones graves, dolor de pecho, falla en la respiración y dificultad del habla ^4^.

Alrededor del 5% de los pacientes con coronavirus se enferman de manera grave y requieren ingreso en las unidades de cuidados intensivos. Estos pacientes, generalmente, son sometidos a ventilación mecánica, procedimiento delicado que a largo plazo puede evidenciar un desarrollo de infecciones bacterianas o fúngicas ^1^^,^^5^.

Cuando un paciente se encuentra enfermo de manera crítica, suben los niveles de citocinas pro y antiinflamatorias, lo cual aumenta el riesgo de infecciones fúngicas, como aspergilosis pulmonar o candidiasis ^2^. La COVID-19 severa está ligada a una alteración de la regulación inmunitaria, que interfiere en las respuestas tanto de las células T auxiliares 2 (Th2) como de las Th1, incluida la liberación de citocinas, lo que contribuye a la formación de una patología pulmonar y promueve una proliferación microbiana y una infección sistémica posterior ^5^.

### Pacientes portadores de prótesis removibles y *Candida albicans*

**Figura 1:**
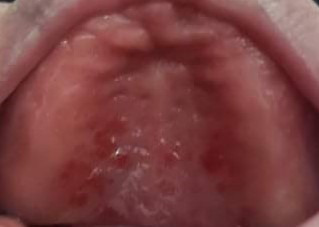
Estomatitis herpética paciente edéntulo total

El edentulismo es la falta de piezas dentales parcial o total, alrededor de 158 millones de personas. El 2,3% de la población a nivel mundial tienen pérdida severa de dientes ^6^. Frente a la alta prevalencia de edentulismo a nivel mundial, existen diversos procedimientos rehabilitadores para devolver la función y la estética perdidas; uno de ellos es el uso de prótesis dentales totales y parciales fijas o removibles. Estos elementos artificiales, elaborados en resina acrílica, pueden ser un recipiente para la proliferación de microorganismos, debido a una mala higiene, el propio material protésico, el uso extendido del aparato, su uso constante durante las noches, la inmunosupresión, la disminución en el flujo salival, el mal ajuste protésico y la alimentación a base de carbohidratos ^6^. El hongo de la *Cándida* es el microorganismo más prevalente en estas condiciones bucales, por lo general, se ubica en la mucosa palatina o en el dorso lingual, y se manifiesta como placas blanquecinas en el tejido gingival y las amígdalas ^7^ (Fig. 1)

Cualquier persona puede presentar candidiasis, pero es más común en las de la tercera edad, debido a que pueden presentar un déficit inmunitario más marcado por la presencia de enfermedades sistémicas. Por esta razón, la candidiasis oral en personas sanas es algo simple y tratable, mientras que en los pacientes inmunodeprimidos el cuadro clínico sería más grave y con cierta dificultad de controlar ^7^.

La tasa de *Candida* en pacientes de la tercera edad o adultos mayores que utilizan prótesis dentales bimaxilares pueden llegar al 65%, debido a que el material protésico que recubre la mucosa oral crea el microambiente específico para su proliferación ^8^.

Desde el punto de vista patológico, los procesos infecciosos en la cavidad bucal pueden provocar procesos inflamatorios sistémicos que afectan diversos órganos y empeoran las patologías preexistentes ^8^. Los microorganismos bucales no solo están implicados en el desarrollo de enfermedades, como periodontitis, caries o estomatitis, sino que también pueden aumentar el riesgo de enfermedades sistémicas, como neumonía por asfixia, infecciones gastrointestinales, sepsis, pleuresía y endocarditis bacteriana ^8^.

Los hongos generadores de *Candida* se caracterizan por ser organismos oportunistas y son detonadores de infecciones nasocomiales, capaces de agravar las condiciones sistémicas preexistentes en un paciente. Los pacientes internados en casas de salud presentan mayor predisposición a la candidiasis oral debido a las condiciones microambientales y sistémicas que alteran la microbiota oral ^9^.

### *Candida albicans* y COVID-19

Cualquier persona, independientemente de su edad, llega a ser blanco fácil de la COVID-19, aunque cabe recalcar que los adultos mayores y los pacientes con enfermedades sistémicas son los que presentan un mayor riesgo de que esta patología agrave de manera desmesurada sus posibles síntomas ^10^.

Las personas de edad avanzada son también el grupo con mayor probabilidad de tener infecciones fúngicas, debido a que presentan una menor inmunidad, generalmente ligada a alteraciones sistémicas ^10^.

Todo tipo de patología crónica en desarrollo produce una inmunodepresión marcada. Si a esto se suma la presencia de una infección con SARS CoV-2, el paciente es muy susceptible al desarrollo de linfoadenopatías que requieren tratamientos que pueden aumentar su predisposición a adquirir infecciones fúngicas, lo que provoca una hospitalización inmediata, con uso de antibióticos, corticoides y procesos de respiración mecánica ^11^^,^^12^.

Se ha manifestado que los procesos fúngicos de *Cándida* en el tracto respiratorio, en pacientes inmunodeprimidos sometidos a respiración mecánica de larga duración, producen un mayor riesgo de enfermedad y una mayor predisposición a padecer neumonía, esto asociado con una mayor duración de la estancia en la UCI ^13^.

Hasta la actualidad no se ha tomado en cuenta de manera muy puntual la prevalencia de infecciones fúngicas en personas contagiadas de coronavirus; sin embargo, en la mayoría de los casos, estos pacientes, al tener una condición de salud desfavorable, necesitan tratamientos que pueden afectar de manera severa el sistema inmune y predisponerlo a una coinfección por candidiasis orofaríngea (CO) ^13^.

En un 80% de los casos de CO, el sistema inmune del paciente se encuentra débil y presenta alteraciones de gusto, síndrome de boca ardiente y disfagia. El hongo principalmente causante es la *Candida albicans*^14^, que en caso de no ser tratado o que el tratamiento haya sido ineficaz, puede propagarse de manera regional hasta el esófago o de manera sistémica hasta el torrente sanguíneo o el tracto intestinal, y es capaz de producir candidemia, mal que posee una alta tasa de mortalidad. Por lo tanto, el descubrimiento oportuno de CO en personas que padecen coronavirus es importante para aplicar un tratamiento eficaz ^14^.

Un estudio identificó la presencia de *Candida albicans* en un 71% de los pacientes infectados por SARS CoV-2, de los cuales un 56,8% eran mayores de 49 años. Dicho estudio determinó que mantener controlada la progresión y la gravedad del estado del paciente puede evitar el desarrollo mortal de la enfermedad, especialmente en pacientes vulnerables y de edad avanzada ^15^.

### Mucosa oral y COVID-19

Estudios recientes indican que la enzima convertidora de angiotensina 2 (ACE2) es utilizada como camino de entrada del COVID-19 a la célula huésped, lo que provoca la infección final. Este ACE2 rodea el tejido oral y gingival, además de las células epiteliales de las glándulas productoras de saliva, y hace que la cavidad bucal actúe como anfitriona en el desarrollo de la infección por SARS-CoV-2 ^16^^,^^17^. Debido al comportamiento de la enfermedad, el fluido salival puede llegar a infectarse por los tractos respiratorios tanto inferior como superior, por la presencia de SARS-CoV-2 en la sangre, y puede acceder a la cavidad bucal a través del fluido crevicular presente en la encía de recubrimiento y por infección de las diferentes glándulas salivales ^18^.

Existen factores predisponentes para el desarrollo de COVID-19, entre ellas factores generales como la edad o condiciones sistémicas y factores locales, entre ellas la xerostomía y la mala higiene bucal ^19^^,^^20^.

Después del análisis de los 65 artículos científicos que cumplieron los criterios de búsqueda, se expone en la Tabla 1 la relación que hay entre la candidiasis oral y los pacientes con COVID-19. 

**Tabla 1:** Relación de estudios sobre pacientes COVID-19 con coinfección de candidiasis oral

Estudio	Pacientes COVID-19 + Candidiasis oral	UCI / Uso de ventilador artificial	Complicaciones	Observaciones
Jerônimo *et al*., 2022	n/a	X	Neumonía (5,2%)	El 75% de las muestras respiratorias mostró la presencia de *Candida albicans*
Segrelles *et al*., 2021	31	X	NO	Las muestras procedentes de fluidos nasorespiratorios mostraron la presencia de *Candida albicans*. El tiempo en UCI de estos pacientes fue mayor en comparación con quienes no presentaron el hongo.
Ohashi *et al*., 2021	1	X	Neumonía	Se determinó que el factor de riesgo de los pacientes con COVID-19 con coinfección por *Candida albicans* fue el uso prolongado de antibióticos.

## DISCUSIÓN

La presente revisión tuvo como objetivo relacionar la presencia de *Candida albicans* como un factor agravante en pacientes con COVID-19, siendo pocos los estudios clínicos que analizan esta coinfección en un mismo individuo.

La candidiasis oral, cuya cepa predominante es la *Candida albicans*, se presenta en pacientes portadores de prótesis dentales removibles ^1^. Este hongo se desarrolla de manera oportunista aprovechándose de un medio ambiente propicio para su evolución como es la cavidad bucal, ya sea por una higiene deficiente o por una inmunosupresión del sistema, como en el caso de pacientes graves por SARS-CoV-2 ^2^.

Las lesiones intraorales provocadas por candidiasis en pacientes diagnosticados previamente con coronavirus se ubicarán principalmente en la superficie donde descansa el aparato protésico ^3^. La presencia de *Candida albicans* por sí sola no afecta en gran medida a quien lo porta; sin embargo, y debido a que la probabilidad de contagio con COVID-19 es bastante alta en la actualidad, el sistema inmune de esta coinfección puede hacer que la persona que lo padezca decaiga y da oportunidad a que dicha infección fúngica forme parte de una “superinfección” capaz de aumentar tanto la morbilidad como la mortalidad ^4^^,^^19^^-^^25^. Esto se debe a que, una vez adquirida la infección fúngica, el coronavirus se encargará de alterar el equilibrio de la microbiota, lo que permite una proliferación más acelerada y marcada sobre la mucosa oral ^8^.

La complicación más evidente en esta coinfección es la neumonía, debido a que los pacientes son internados durante mayor tiempo en unidades de cuidados intensivos y requieren ventilación asistida e intubación, lo que hace estos cuadros sintomáticos más graves y complejos de tratar ^1^^,^^2^^,^^4^.

La edad y la mala higiene oral son factores de riesgo para la colonización bacteriana orofaringea ^7^^,^^14^^,^^19^^,^^21^. Además, en pacientes con trastornos inmunoreguladores o con consumo común de antibióticos, puede hacer que se presente la coinfección y agrave la COVID-19 ^4^.

La cavidad oral es el portal de ingreso para la infección por SARS-CoV-2 por la afinidad del virus con los receptores ECA2 presentes en la mucosa oral, la lengua y las glándulas salivales ^3^^,^^5^. Esto hace necesario combinar el tratamiento vírico con el fúngico para controlar la “superinfección” ^4^. En la actualidad, la resistencia a los antifúngicos ha aumentado y se necesitan más investigaciones para encontrar nuevos productos efectivos para tratar la candidiasis oral ^7^.

No se sabe si la presencia de *Candida albicans* en el paciente con COVID-19 causa una mortalidad excesiva o simplemente es un indicador de la gravedad de la infección por el virus SARS-CoV-2; por lo tanto, se hace necesario realizar más estudios que determinen la verdadera carga necesaria de *Candida albicans* para agravar la infección por COVID-19 ^6^^,^^10^^,^^25^^-^^28^.

## CONCLUSIONES

La candidiasis oral se relaciona de manera directa con la utilización de prótesis dentales, lo que afecta de manera negativa a pacientes con infección de COVID-19 y aumenta el riesgo de ingreso a UCI para el uso de ventiladores artificiales. Este efecto se debe a la desregularización inmunológica del paciente o al uso constante de antibióticos.

Es importante el control temprano de la infección por *Candida albicans* para evitar complicaciones en pacientes infectados con SARS-CoV-2.

## Fuente de financiamiento:

Este trabajo no fuentes de financiamiento
